# Comprehensive analysis of mitochondrial unfolded protein response related genes for prognosis and therapeutic response in pancreatic cancer

**DOI:** 10.3389/fimmu.2026.1717925

**Published:** 2026-02-05

**Authors:** Chengqing Li, Cuiling Lu, Zheng Ma, Ting Zhao, Mingming Xiao, Zhonglei Xu, Zhenxing Sun, Qihao Wu, Lei Wang, Liang Zhao

**Affiliations:** 1 Pancreas Center, Tianjin Medical University Cancer Institute and Hospital, National Clinical Research Center for Cancer, State Key Laboratory of Druggability Evaluation and Systematic Translational Medicine, Tianjin Key Laboratory of Digestive Cancer, Tianjin’s Clinical Research Center for Cancer, Tianjin, China; 2 Department of Pancreatic Surgery, General Surgery, Qilu Hospital of Shandong University, Jinan, China; 3 Ganjiang Chinese Medicine Innovation Center, Nanchang, China

**Keywords:** drug sensitivity, machine learning, mitochondrial unfolded protein response, nomogram, pancreatic cancer, prognosis

## Abstract

**Background:**

Pancreatic cancer (PC) is a highly aggressive malignancy of the digestive system, with an extremely poor prognosis. The mitochondrial unfolded protein response (UPR^mt^) can maintain mitochondrial homeostasis and promote tumor progression and chemotherapy resistance. Nevertheless, the functions of UPR^mt^-related genes (MRGs) in PC remain undefined.

**Methods:**

Gene expression data were obtained from TCGA, GEO, and CPTAC databases. Consensus clustering was performed based on MRGs, with subsequent evaluation of immune infiltration patterns across clusters. Prognostic MRGs were identified using three machine learning algorithms: LASSO regression, Random Survival Forest (RSF), and Extreme Gradient Boosting (XGBoost), combined with Cox regression analysis to establish a MRGs risk score (MRS). Quantitative real-time PCR (qRT-PCR) and western blotting were employed to validate potential mechanisms. Drug sensitivity profiling distinguished therapeutic responses between risk groups. Finally, we developed an MRS-based prognostic nomogram and validated it in multiple cohorts.

**Results:**

PC patients were stratified into two distinct UPR^mt^ clusters with notable differences in overall survival (OS) and immune cell infiltration. Through screening, we established a novel MRS based on three prognostic core genes (CAT, CEBPB, and PRKN). High MRS patients showed significantly poorer OS compared to low MRS patients. We observed marked differences in drug sensitivity between subgroups and further predicted potential therapeutic agents targeting MRS. The prognostic nomogram based on MRS demonstrated strong predictive accuracy for 1-, 2-, and 3-year OS across both training and validation PC cohorts. Furthermore, western blot analysis preliminarily validated the potential association between UPR^mt^ and both P53 signaling and glycolysis pathways.

**Conclusion:**

Our study systematically characterizes the prognostic and therapeutic implications of MRGs in PC, establishing a 3-gene MRS capable of reliably predicting OS in PC patients and exploring UPR^mt^ potential oncogenic mechanisms. These findings provide a valuable reference for individualized therapeutic strategies in PC management.

## Introduction

Pancreatic cancer (PC) is an aggressively malignant neoplasm of the digestive system, characterized by difficulties in early diagnosis, high rates of recurrence and metastasis, and frequent chemotherapy resistance, posing a substantial global burden in cancer management (1, 2). Although therapeutic approaches have improved significantly, the overall survival (OS) of PC patients remains poor, with a 5-year survival rate of only 13%, making PC the sixth leading cause of cancer-related deaths worldwide (1, 3). Projections suggest that by 2030, PC will rank as the second leading cause of cancer-related deaths in the United States (4). With the growing understanding of cancer immunology and metabolism, immunotherapy and metabolic targeting have emerged as promising therapeutic strategies (5). PC exhibits a profoundly immunosuppressive tumor microenvironment and extensive metabolic reprogramming, features that synergistically drive tumor invasiveness and therapy resistance (6, 7). These characteristics highlight the urgent need to develop novel therapeutic strategies that target immune and metabolic pathways.

Mitochondria, serving as the energy metabolism hub of eukaryotic cells, play a pivotal role in essential biological processes such as oxidative phosphorylation, apoptosis, and calcium homeostasis (8, 9). When mitochondria are compromised due to factors such as energy metabolism imbalance, mitochondrial DNA damage, reactive oxygen species accumulation (ROS), or protein misfolding, a cascade of adaptive and pathological responses will be triggered, collectively termed mitochondrial stress (10, 11). The mitochondrial unfolded protein response (UPR^mt^) serves as an evolutionarily conserved protective mechanism during mitochondrial stress, triggered specifically by the aberrant buildup of unfolded or misfolded proteins within the mitochondrial matrix (12, 13). Through mitochondrial-nuclear retrograde signaling, UPR^mt^ upregulates the expression of mitochondrial chaperones (such as HSP60, HSP10) and proteases (such as CLPP, LONP1) to clear misfolded proteins, and restore mitochondrial function (13, 14). The hypoxic and oxidative stress microenvironment of PC induces mitochondrial stress, which in turn activates the UPR^mt^ to maintain mitochondrial homeostasis and promote tumor viability (15–18). Activation of the UPR^mt^ has been shown to promote tumor invasion, metastasis, chemotherapy resistance, and immune escape (12, 15, 19). Therefore, targeting UPR^mt^ to disrupt mitochondrial homeostasis in tumor cells could present a promising therapeutic strategy (20).

Although the role of UPR^mt^ has been partially validated in certain malignancies (21, 22), a comprehensive analysis of UPR^mt^-related genes (MRGs) in PC is still lacking. Therefore, we conducted an in-depth analysis of MRGs in PC, systematically investigating their expression patterns, prognostic value, immune microenvironment associations, and drug sensitivity profiles. Furthermore, we identified three core prognostic MRGs through three machine learning methods and construct a MRGs risk score (MRS). The predictive robustness of MRS was rigorously validated across multiple independent cohorts. Our findings not only underscore the pivotal role of the UPR^mt^ in PC progression but also offer novel molecular biomarkers for prognosis assessment and potential therapeutic targets for personalized treatment strategies.

## Materials and methods

### Data download and processing

RNA sequencing data of PC, along with patient-matched clinical data, were acquired from three sources: (i) The Cancer Genome Atlas-Pancreatic Adenocarcinoma (TCGA-PAAD) cohort was extracted from the UCSC Xena platform (http://xena.ucsc.edu/) (23); (ii) The PC related datasets GSE224564 (24) was retrieved from the Gene Expression Omnibus (GEO) database (http://www.ncbi.nlm.nih.gov/geo); (iii) The Clinical Proteomics Tumor Analysis Consortium-Pancreatic Ductal Adenocarcinoma Discovery Study (CPTAC-PDAC) cohort (25) was obtained from the LinkedOmics platform (http://www.linkedomics.org/) (26). Samples with missing survival data were excluded. After screening, TCGA-PAAD cohort contained 177 tumor and 4 normal samples, GSE224564 cohort contained 175 tumor samples, and CPTAC-PDAC cohort contained 135 tumor and 21 normal samples. Given the limited number of normal samples in TCGA-PAAD, we supplemented 167 normal pancreatic samples by obtaining the combined and normalized TCGA and Genotype-Tissue Expression (GTEx) data for subsequent difference expression analysis from UCSC Xena platform. Detailed data sources were provided in **Supplementary Table 1**. Both single nucleotide variants (SNV) and copy number variation (CNV) data were obtained from TCGA database. SNV data were processed using the “maftools” R package (27), while CNV data were analyzed with GISTIC 2.0 (28). In this study, the TCGA-PAAD cohort was employed as the training set, with GSE224564 cohort and CPTAC-PDAC cohort serving as the validation sets.

### Definition of MRGs

First, the “Mitochondrial Unfolded Protein Response” pathway (R-HSA-9841251) was retrieved from the Reactome database (29) (http://reactome.org/), and its associated gene set was extracted. Subsequently, through further integration of prior relevant studies (12, 21, 30, 31), we curated a total of 43 mitochondrial MRGs. Detailed information of MRGs is summarized in **Supplementary Table 2**.

### Enrichment analysis

Functional annotation of MRGs was performed using Gene Ontology (GO), Kyoto Encyclopedia of Genes and Genomes (KEGG) and Reactome pathway enrichment analyses implemented in the “clusterProfiler” (32) and “ReactomePA” (33) R packages.

### Consensus clustering of MRGs

Consensus clustering based on MRGs was conducted by the “ConsensusClusterPlus” (34) R package for stratifying PC patients into subgroups. The clustering process was performed with a maximum cluster number set to 6, and 80% of samples were randomly drawn in 500 replicates to enhance the reliability of the consensus clustering. We comprehensively evaluated the consensus cumulative distribution function (CDF), delta area plot, and consensus matrix results to identify the optimal cluster number. Uniform Manifold Approximation and Projection (UMAP) was applied to visualize the distribution of samples and assess the effectiveness of consensus clustering. Furthermore, GSVA (35) was employed to assess differential activity of 50 canonical Hallmark pathways (from the MSigDB database, http://www.gsea-msigdb.org/gsea/msigdb) between subgroups, and Kaplan-Meier analysis was employed to assess differences in OS across groups.

### Immune infiltration analysis

To comprehensively evaluate the immune landscape between the two subgroups, we employed multiple algorithms, including single-sample Gene Set Enrichment Analysis (ssGSEA), CIBERSORT, xCell, EPIC, and ESTIMATE. Specifically, ssGSEA was performed to calculate the infiltration scores of 28 immune cell types based on predefined immune-related gene sets (36), while xCell estimated the relative abundance of 64 immune and stromal cell types by integrating cell type-specific gene signatures (37). CIBERSORT (38) and EPIC (39) further quantified the proportions of diverse immune and stromal cell types within the tumor microenvironment, with results visualized using stacked bar plots. Additionally, ESTIMATE was employed to predict stromal and immune cell infiltration levels in tumor tissues to infer tumor purity (40). Finally, the expression levels of common immune checkpoint genes were compared across subgroups.

### Prognostic MRG screening and MRS construction

First, preliminary screening of potential prognostic MRGs (P<0.05) was conducted using univariate Cox regression. Subsequently, these potential prognostic MRGs were further selected through three machine learning methods: Least Absolute Shrinkage and Selection Operator (LASSO) regression, Random Survival Forest(RSF), Extreme Gradient Boosting (XGBoost). The intersection of the selected MRGs from all three methods was then subjected to multivariate Cox regression, identifying core prognostic MRGs for construction of MRS. The MRS was calculated according to the following formula:


$$MRS=\sum_{n=1}^{\infty }[coefficient(n)*expression\;of\;m\text{RNA}(n)]$$

Patients were divided into high- and low-MRS subgroups using the median MRS value, and OS differences were compared by Kaplan-Meier survival curves. Furthermore, the “survminer” R package was employed to identify the optimal cutoff value for each cohort, and the prognostic stability of the MRS was further validated by comparing survival differences under the optimal stratification. The relationship of MRS with both clinicopathologic features and pathway activity profiles was analyzed. Further validation for the expression levels of core MRGs was performed through the TNMplot database (41).

### Drug sensitivity prediction

Drug sensitivity was predicted for each subgroup according to the Genomics of Drug Sensitivity in Cancer (GDSC) database using the R package “pRRophetic” (42). The half-maximal inhibitory concentration (IC_50_) values for standard chemotherapeutic agents were computed and compared across subgroups. The Tumor Immune Dysfunction and Exclusion (TIDE) score was employed to predict the probabilities of immunotherapy responses for patients (43). Potential drug candidates targeting the three core MRGs were predicted using the Drug Signatures Database (DSigDB) within the Enrichr platform (http://maayanlab.cloud/Enrichr/).

### Nomogram construction and validation

Prognostic independent risk factors were selected from clinicopathological characteristics through univariate and multivariate Cox regression analyses in the training cohort. Utilizing these selected prognostic factors, the “rms” R package was employed to develop a nomogram for predicting 1-, 2-, and 3-year OS in PC patients. The performance and reliability of the nomogram were assessed in both training and validation cohorts using time-dependent receiver operating characteristic (ROC) curves and decision curve analysis (DCA).

### Cell culture and transfection

The human pancreatic cancer cell lines (PANC-1 and BxPC-3) were acquired from the American Type Culture Collection (ATCC, Manassas, VA, USA). Cells were cultured in DMEM or RPMI-1640 medium (Gibco) which was supplemented with 10% fetal bovine serum (ExCell, FSP500) and 1% penicillin/streptomycin (Solarbio, P1400) at 37°C in a 5% CO_2_ incubator. All cell lines were authenticated by short tandem repeat (STR) profiling and routinely tested for mycoplasma contamination using the MycAway Plus-Color One-Step Mycoplasma Detection Kit (Yeasen, 40612ES08) to ensure experimental reliability.

The plasmids used in this study were obtained from MiaoLingBio (China), including CEBPB-shRNA1 (MiaoLingBio, P39946), CEBPB-shRNA2 (MiaoLingBio, P39948), and a Scrambled control (MiaoLingBio, P1717). Transfection of PANC-1 and BxPC-3 cells was performed using the Lipo8000 transfection reagent (Beyotime, C0533) following the manufacturer’s protocols.

### UPR^mt^ activation

Rotenone is a mitochondrial complex I inhibitor that induces mitochondrial stress by promoting ROS production. To induce UPR^mt^, cells at approximately 80% confluency were treated with 1 µM rotenone (MCE, HY-B1756) for 24 hours.

### Quantitative real-time polymerase chain reaction

We extracted total RNA from cells using RNA extraction kit (SparkJade, AC0205-B). Then, cDNA was synthesized from RNA by the reverse transcription kit (GenStar, A230-10). Real-time quantitative polymerase chain reaction (RT-qPCR) was conducted with the 2×RealStar Power SYBR qPCR Mix (GenStar, A311-10). Real-time detection was carried out using the PCR plates (GenStar, HL96112-01), and the relative gene expression levels were calculated using the 2^–ΔΔCT^ method. Three independent replicates were conducted for each experiment. The sequences of all primers were shown in **Supplementary Table 3**.

### Cell viability analysis

After transfection, PANC-1 and BxPC-3 cells were seeded into 96-well plates at a density of 5000 cells per well. At 0, 1, 2, and 3 days post-seeding, 100μL of culture medium containing 10μL of Cell Counting Kit-8 (CCK-8, MeilunBio, MA0218) was added to each well. Following incubation for 1.5 hours at 37°C, the absorbance at 450nm was measured using a microplate reader to evaluate cell viability and proliferation.

### Wound healing assay

PANC-1 and BxPC-3 cells were seeded into 6-well plates at an appropriate density and cultured until confluence. Then, a linear scratch was created across the cell monolayer using a sterile 200 μL pipette tip. Detached cells were gently removed by washing with phosphate-buffered saline, after which serum-free medium was added. Wound closure was monitored and imaged at 0 and 24 hours post-scratch under a microscope.

### Western blotting

Cells were treated and lysed in RIPA buffer (Solarbio, R0010) containing a protease inhibitor (Selleck, B14001). After centrifugation at 12000 g for 15 min at 4°C, the supernatant was collected as total protein. Protein concentration was quantified by the bicinchoninic acid assay (Beyotime, P0009–1 and P0009-2), and all samples were adjusted to equal concentrations. Equal amounts of protein (20–40 μg) were subjected by SDS-PAGE and transferred onto PVDF membranes (Millipore, ISEQ00010). Membranes were blocked with 5% non-fat milk in TBST buffer at room temperature for 1 h and subsequently incubated overnight at 4°C with the primary antibodies against CEBPB (Proteintech, 23431-1-AP), HSP60 (Proteintech, 66041-1-IG), p21 (Proteintech, 10355-1-AP), PKM2 (Proteintech, 15822-1-AP), β-actin (Beijing Ray Antibody Biotech, RM2001), β-tubulin (Beijing Ray Antibody Biotech, RM2003). Following three washes with TBST (10 min each), membranes were incubated with HRP-conjugated secondary antibodies (Cell Signaling, 7074S and 7076S) for 1 h at room temperature. Protein bands were visualized using an ECL detection reagent (Millipore, WBKLS0500) and imaged with a chemiluminescence detection system. Three independent replicates were conducted for each experiment.

### Statistical analysis

Data analysis and visualization were conducted by GraphPad Prism (version 10.1.2) and R (version 4.4.1). Based on normality test, intergroup differences were analyzed using either Student’s t-test or Wilcoxon rank-sum test as appropriate. Intergroup comparisons of proportions between two groups were performed using Pearson Chi square or Fisher’s exact tests. Pearson or Spearman correlation coefficients were computed to quantify associations between variables. Kaplan-Meier survival curves with log-rank testing were used to compare survival differences between groups. All statistical tests were two-sided, with a P < 0.05 indicating statistical significance.

## Results

### Characterization of MRGs in pancreatic cancer

**Figure 1:**
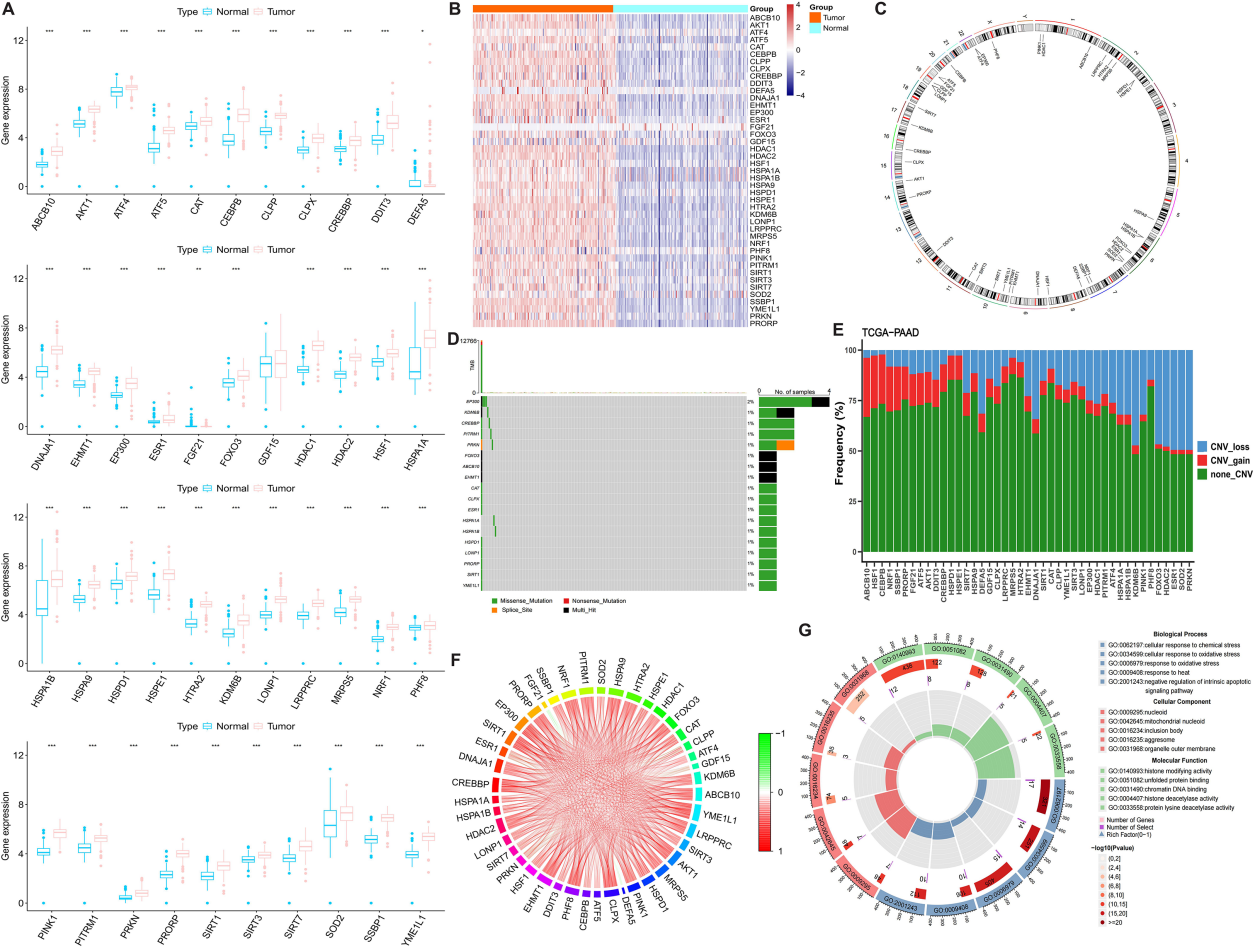
Expression, mutation, and functional analysis of MRGs in pancreatic cancer. **(A)** Boxplots of MRGs expression differences between PC and normal pancreatic tissues. **(B)** Heatmap of MRG expression profiles in PC and normal tissues. **(C)** Genomic distribution of 43 MRGs across chromosomes. **(D)** SNV frequency and types of MRGs. **(E)** CNV landscape of MRGs. **(F)** Correlation of expression levels across 43 MRGs in PC. **(G)** GO enrichment analysis of MRGs. *p < 0.05, **p < 0.01, ***p < 0.001.

A comparative analysis of 43 MRGs across PC and normal pancreatic tissues from the TCGA and GTEx datasets showed that the majority of MRGs exhibited significantly elevated expression in tumors (**Figures 1 A, B**). The chromosomal locations of these 43 MRGs are depicted in **Figure 1C**. SNV of MRGs were identified in 10 patients, with missense mutations being the most prevalent variant type (**Figure 1D**). CNV analysis demonstrated a higher frequency of copy number gain in genes such as ABCB10, HSF1, and CEBPB, while genes like PRKN, SOD2, ESR1, FOXO3, HDAC2, and KDM6B showed a greater frequency of copy number loss (**Figure 1E**). The correlation network diagram further elucidates the expression relationships among MRGs (**Figure 1F**). Enrichment analysis highlighted the participation of these MRGs in several stress response pathways, such as “response to oxidative stress”, “cellular response to chemical stress”, and “response to heat”, among others (**Figure 1G**, **Supplementary Figures 1A, B**).

### Consensus clustering based on MRGs

**Figure 2:**
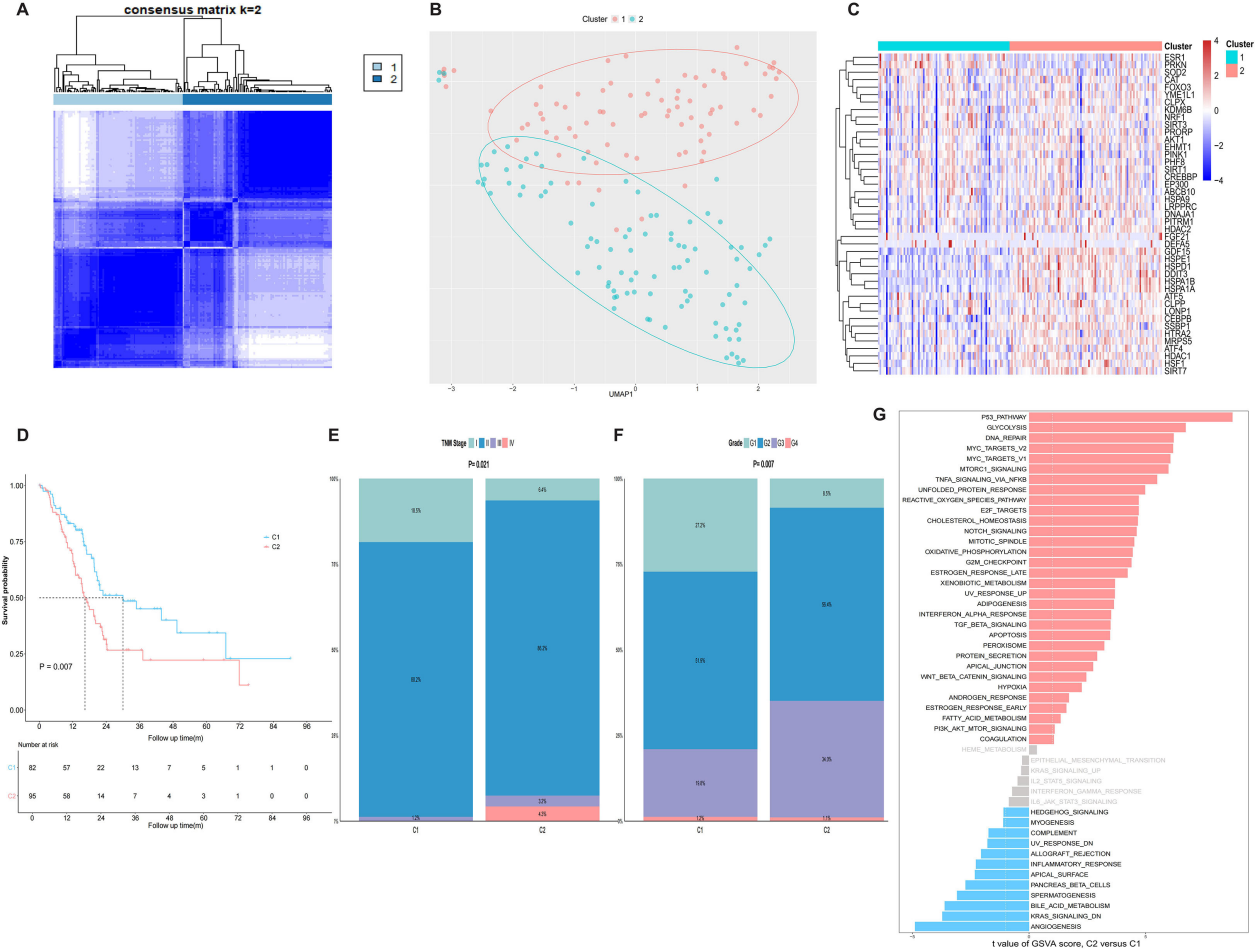
MRG-based consensus clustering identifies molecular subtypes in TCGA-PAAD cohort. **(A)** Consensus matrix heatmap reveals two stable clusters in TCGA-PAAD cohort. **(B)** UMAP analysis showing clear separation between two clusters at k=2. **(C)** Heatmap of MRG expression profiles between two clusters. **(D)** Kaplan-Meier curve for OS between two clusters. **(E)** TNM stage distribution disparity between two clusters. **(F)** Pathological grades distribution disparity between two clusters. **(G)** Comparison of GSVA scores for 50 Hallmark pathway between two clusters.

Consensus clustering based on MRG expression identified K = 2 as the optimal number of clusters (**Figure 2A**, **Supplementary Figures 2A, B**). UMAP demonstrated that consensus clustering successfully distinguished samples with distinct characteristics (**Figure 2B**). Compared to Cluster 1 (C1), multiple MRGs were significantly upregulated in Cluster 2 (C2), suggesting enhanced UPR^mt^ activation in this cluster (**Figure 2C**). Kaplan-Meier analysis revealed that patients in C2 exhibited significantly worse OS than those in C1 (P = 0.007, **Figure 2D**). Additionally, C2 patients presented more advanced TNM stages (P = 0.021, **Figure 2E**) and higher pathological grades (P = 0.007, **Figure 2F**). Activity assessment of the 50 canonical Hallmark pathways across clusters revealed that pathways such as “P53 PATHWAY”, “GLYCOLYSIS” and “DNA REPAIR” were significantly activated in C2, while others like “ANGIOGENESIS”, “KRAS SIGNALING DN” and “BILE ACID METABOLISM” displayed reduced activity (**Figure 2G**).

### Immune infiltration and immune molecular signatures between clusters

**Figure 3:**
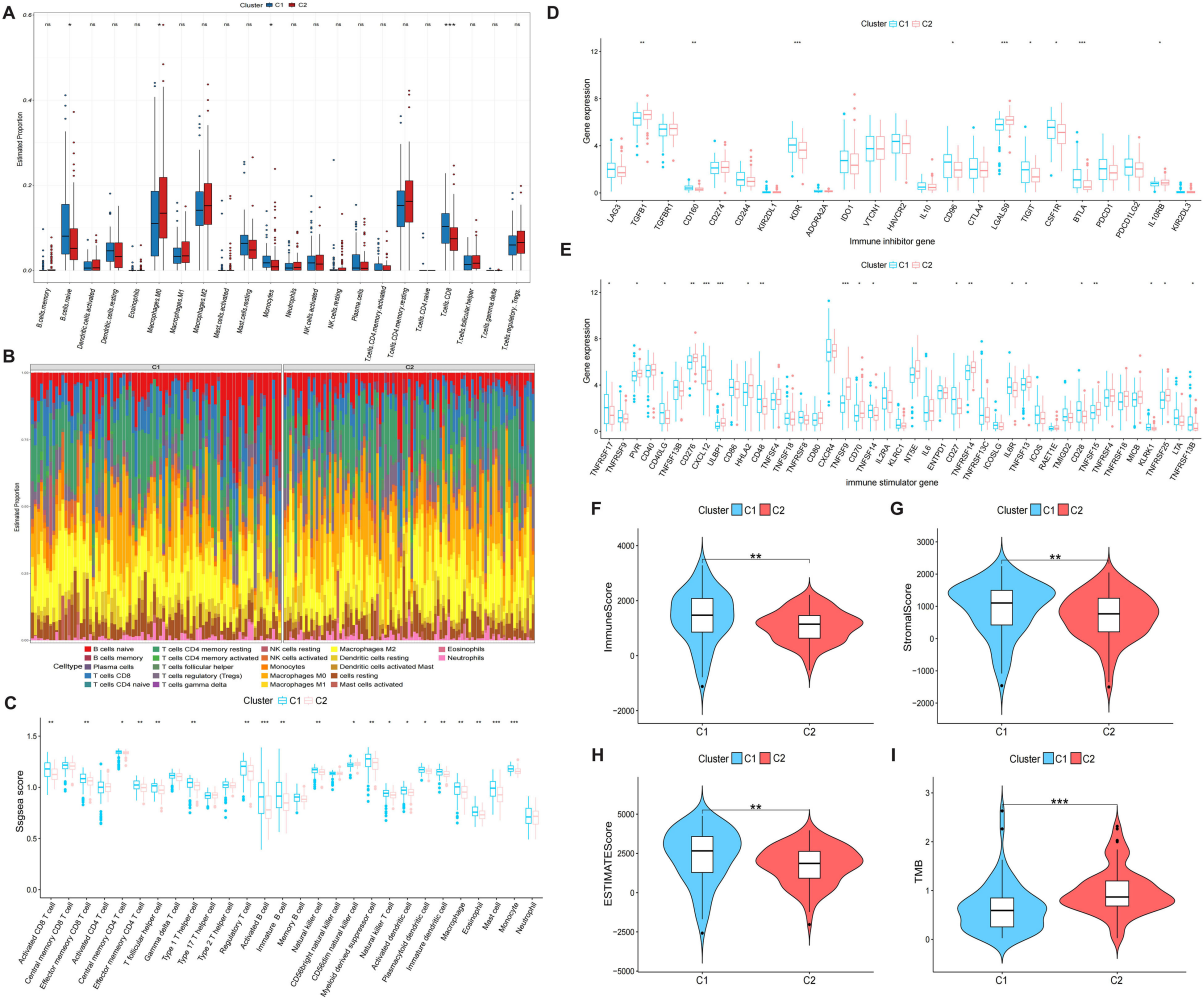
Immunocyte infiltration analysis between two clusters. **(A)** Box plot comparing immune infiltration levels between two clusters using CIBERSORT algorithm. **(B)** Proportional infiltration of immune cells in each PC patient. **(C)** Box plot comparing immune infiltration levels between two clusters using ssGSEA algorithm. **(D, E)** Box plots showing expression levels of immunoinhibitor **(D)** and immunostimulator genes **(E)** between two clusters. **(F–I)** Comparative analysis of Immune Score **(F)**, Stromal Score **(G)**, ESTIMATE Score **(H)**, and TMB **(I)** across clusters.*p < 0.05, **p < 0.01, ***p < 0.001.

CIBERSORT analysis revealed significantly higher proportions of B cells (naive), monocytes, and CD8+ T cells in C1 compared to C2 (**Figure 3A**). The immune cell distribution across the two clusters is comprehensively illustrated in **Figure 3B**. The results of ssGSEA revealed that C1 exhibited higher enrichment levels in most immune cell types compared to C2 (**Figure 3C**). The xCell and EPIC analyses further corroborated these findings, revealing a significantly lower proportion of immune and stromal cells in C2 patients, indicative of a “cold” immune microenvironment (**Supplementary Figures 3A–D**). Additional analysis of immune inhibitor and immune stimulator gene expression between the two clusters revealed significant differences (**Figures 3D, E**). Compared with cluster C1, cluster C2 exhibited elevated expression of TGFB1, LGALS9, IL10RB, PVR, CD276, ULBP1, HHLA2, TNFSF9, CD70, NT5E, TNFRSF14, TNFSF13, TNFSF15, and TNFRSF25, while expression levels of CD160, KDR, CD96, TIGIT, CSF1R, BTLA, TNFRSF17, CD40LG, CXCL12, CD48, TNFSF14, CD27, IL6R, CD28, KLRK1, and TNFRSF13B were reduced. C1 exhibited significantly elevated Immune Score, Stromal Score, and ESTIMATE Score compared to C2 (P < 0.05, **Figures 3F–H**). This indicates that C1 exhibits greater immune cell infiltration, more abundant stromal components, and consequently lower proportions of malignant cells. Furthermore, C1 displayed a significantly lower TMB compared to C2 (**Figure 3I**).

### Development and validation of the MRS

**Figure 4:**
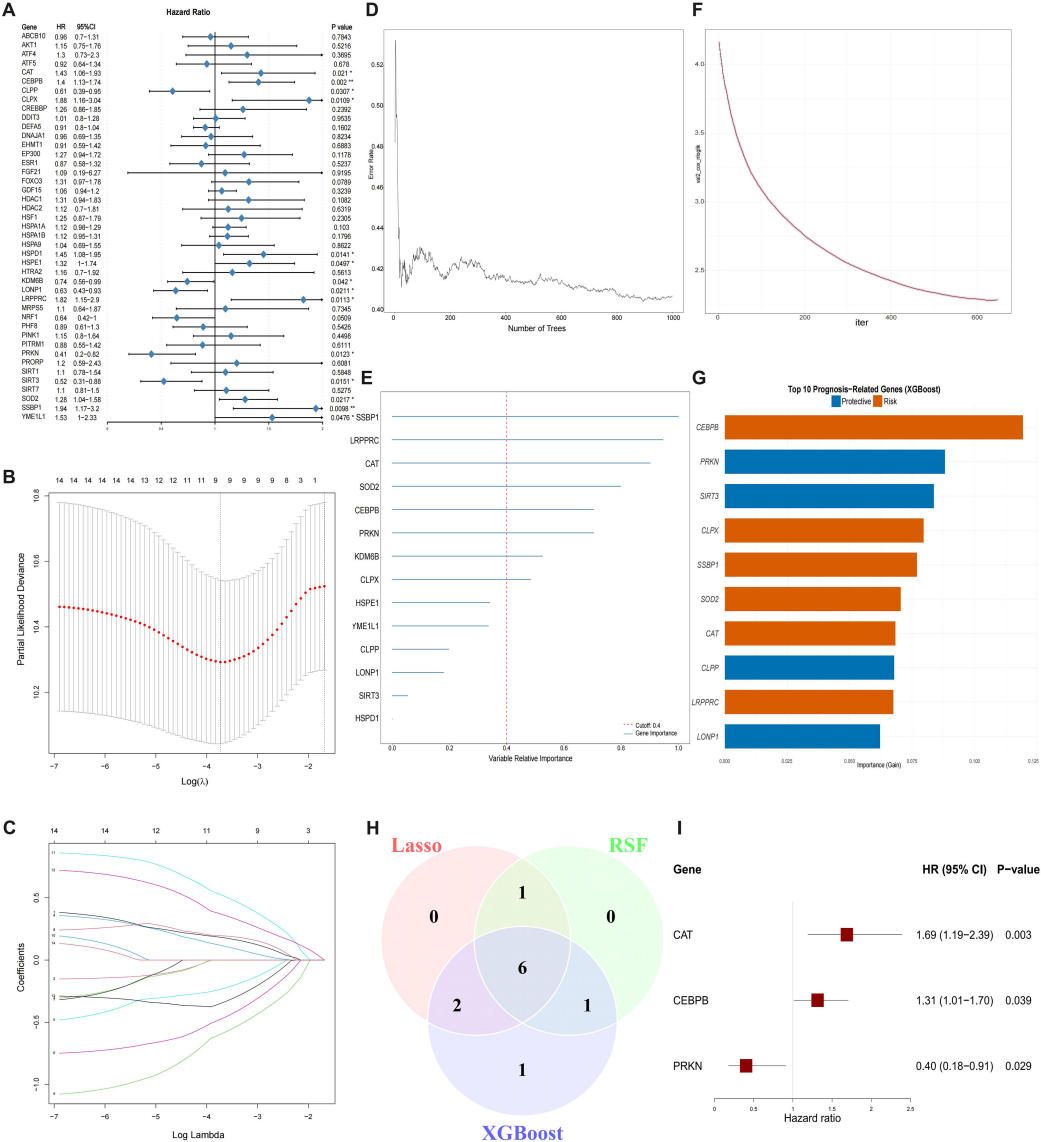
Identification of core prognosis MRGs. **(A)** Forest plot of univariate Cox regression analysis for MRGs. **(B, C)** LASSO regression was performed to screen prognostic MRGs: **(B)** coefficient profiles plot; **(C)** optimum parameter (lambda) selection. **(D, E)** RSF was performed to screen prognostic MRGs: **(D)** relationship of error rate to the number of decision trees; **(E)** relative importance of MRGs. **(F, G)** XGBoost was performed to screen prognostic MRGs:**(F)** training loss convergence; **(G)** top 10 MRGs based on importance. **(H)** Venn diagram of overlapping prognostic MRGs identified by three machine learning algorithms. **(I)** Forest plot of multivariate Cox regression for three core MRGs.

To identify prognostic MRGs in PC, univariate Cox regression analysis was initially conducted on the TCGA-PAAD cohort, leading to the selection of 14 prognosis-related MRGs (P < 0.05), including SSBP1, SOD2, HSPE1, CAT, CLPP, KDM6B, YME1L1, LRPPRC, SIRT3, HSPD1, CLPX, CEBPB, PRKN, LONP1 (**Figure 4A**). We subsequently utilized three independent machine learning algorithms to further screen prognostic MRGs. LASSO regression identified 9 MRGs with non-zero coefficients (**Figures 4B, C**). RSF highlighted 8 MRGs with a variable relative importance greater than 0.4 (**Figures 4D, E**). Through XGBoost, the top 10 key prognostic MRGs were selected (**Figures 4F, G**). The intersection of the results from LASSO, RSF, and XGBoost identified 6 MRGs (CAT, CEBPB, CLPX, LRPPRC, PRKN, SSBP1) (**Figure 4H**). Finally, a multivariate Cox regression was conducted to develop a 3-MRGs prognostic MRS (**Figure 4I**). The MRS was computed according to the formula: MRS = 0.5236 * CAT + 0.2738 * CEBPB - 0.9069 * PRKN.

**Figure 5:**
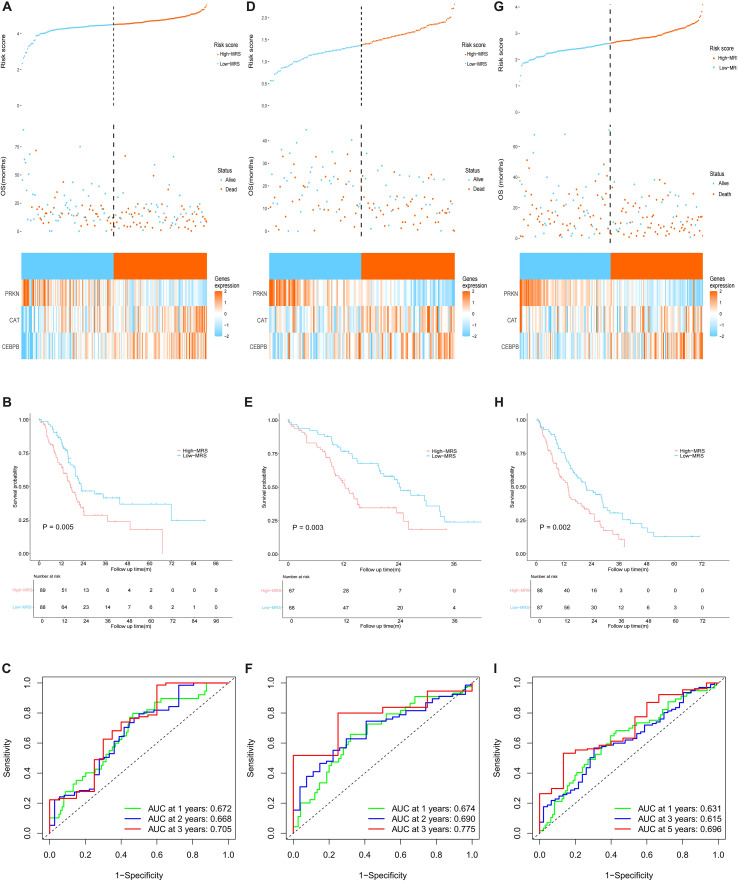
Prognostic value of MRS. **(A–C)** Prognostic value of MRS in TCGA-PAAD cohort: **(A)** relation between MRS And survival status, with heatmap showing expression of three core MRGs; **(B)** Kaplan-Meier survival curves stratified by MRS; **(C)** time-dependent ROC curves assessing 1-, 2-, and 3-year OS. **(D–F)** Prognostic value of MRS in CPTAC-PADC cohort: **(D)** the relation between MRS And survival status, with heatmap showing expression of three core MRGs; **(E)** Kaplan-Meier survival curves stratified by MRS; **(F)** time-dependent ROC curves assessing 1-, 2-, and 3-year OS. **(G–I)** Prognostic value of MRS in GSE224564 cohort: **(G)** the relation between MRS And survival status, with heatmap showing expression of three core MRGs; **(H)** Kaplan-Meier survival curves stratified by MRS; **(I)** time-dependent ROC curves assessing 1-, 2-, and 3-year OS.

Patients were stratified into high- and low-MRS groups using the median cutoff. Subsequently, the prognostic value of MRS and the expression patterns of three core MRGs were evaluated. The high-MRS group exhibited shorter OS and more frequent death events in the TCGA-PAAD cohort (**Figure 5A**). CAT and CEBPB showed elevated expression in high-MRS group, while PRKN showed reduced expression (**Figure 5A**). Kaplan-Meier survival curve demonstrated a significantly shorter OS for high-MRS patients compared to low-MRS patients (P = 0.005, **Figure 5B**). The area under the ROC curve (AUC) for predicting 1-, 2-, and 3-year survival were 0.672, 0.668, and 0.705, respectively, indicating that the MRS exhibits good predictive performance (**Figure 5C**). Further validation in the CPTAC-PDAC cohort (**Figures 5D–F**) and GSE224564 cohort (**Figures 5G–I**) confirmed these findings. In the validation cohort, the high-MRS group also exhibited significantly shorter OS, with expression trends of the three core MRGs being consistent with those observed in the training cohort (P < 0.05, **Figures 5D–I**). In addition, stratification based on the optimal cutoff value was performed to further validate the prognostic value of the MRS, revealing that patients with high MRS still exhibited significantly worse overall survival than those with low MRS (**Supplementary Figures 4A–C**). Collectively, these findings confirmed the MRS as a reliable predictor of OS in PC patients.

### Association between MRS and clinicopathological characteristics

**Figure 6:**
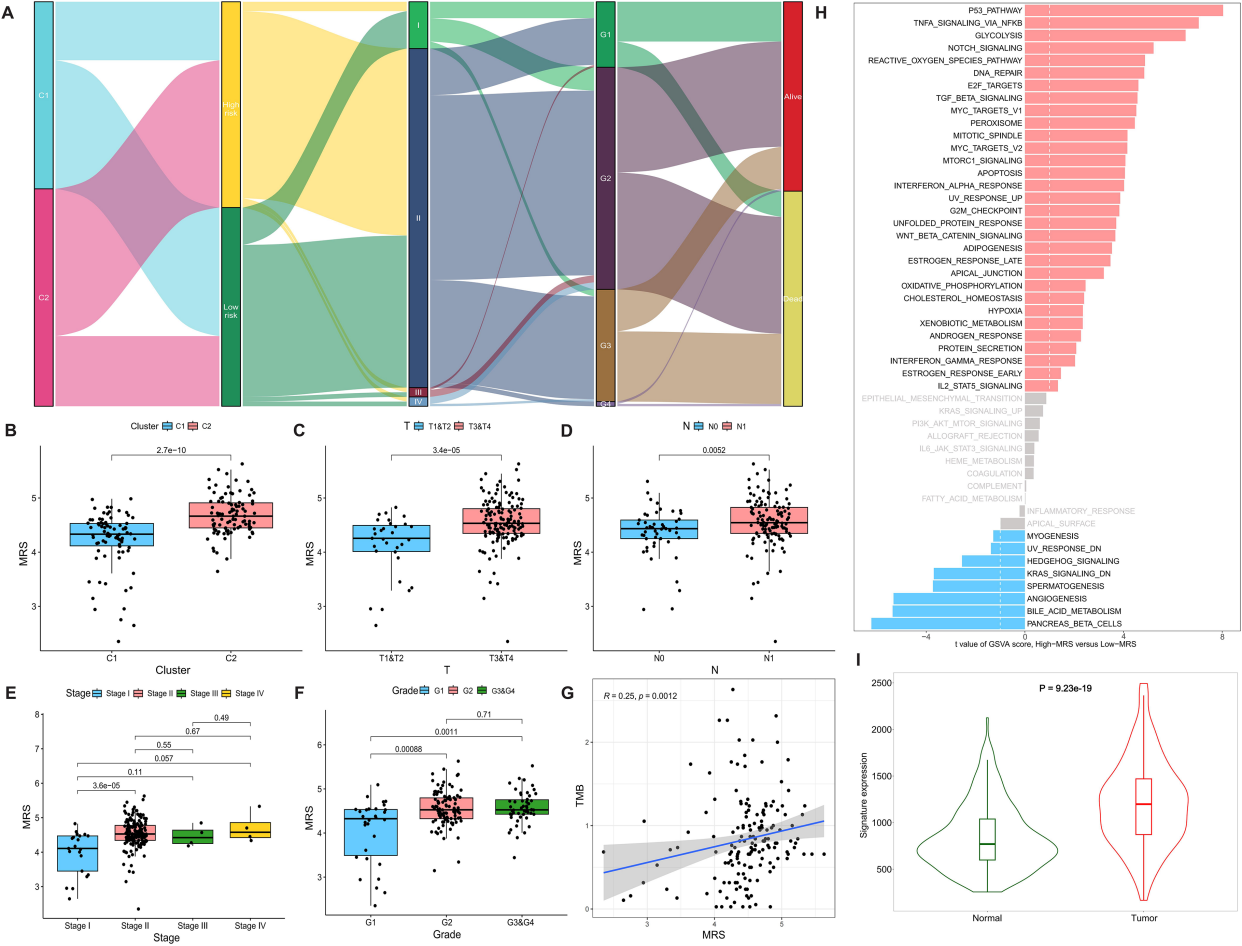
Association between MRS and clinicopathological characteristics. **(A)** Sankey diagram showing relationships among MRS, clusters, tumor stages, pathological grades, and survival status. **(B–F)** MRS distribution across different clusters **(B)**, T stages **(C)**, N stages **(D)**, tumor stages **(E)**, and pathological grades **(F)**. **(G)** Correlation between MRS and TMB. **(H)** Comparison of GSVA scores for 50 Hallmark pathways between high- and low-MRS groups. **(I)** Differential Expression of MRS between PC and normal tissues in TNMplot database.

Analysis of the association between MRS and clinicopathological features demonstrated that MRS was significantly higher in C2 relative to C1, and was associated with tumor stage, pathological grade, and survival events (**Figures 6A, B**). MRS was significantly reduced in tumors with lower T stage (**Figure 6C**), lower N stage (**Figure 6D**), early stage (stage I) (**Figure 6E**), and well-differentiated histology (Grade 1)(**Figure 6F**). Moreover, MRS showed a significant positive correlation with TMB (P = 0.001, **Figure 6G**). The GSVA algorithm assessed differences in activity levels of 50 Hallmark pathways in high-MRS groups relative to low-MRS groups. The results demonstrated that the activity of pathways such as “P53 PATHWAY”, “TNFA SIGNALING VIA NFKB”, and “GLYCOLYSIS” was elevated in high-MRS group, whereas pathways such as “PANCREAS BETA CELLS”, “BILE ACID METABOLISM” and “ANGIOGENESIS” showed a reduced activity (**Figure 6H**). These findings were similar to those obtained from GSVA analysis after clustering based on MRGs (**Figure 2G**), suggesting that MRS could also effectively reflect the activation of UPR^mt^. TNMplot database showed a significantly higher combined expression of three core MRGs in PC relative to normal tissues (P < 0.001, **Figure 6I**).

### Exploring potential therapeutic drugs based on MRS

**Figure 7:**
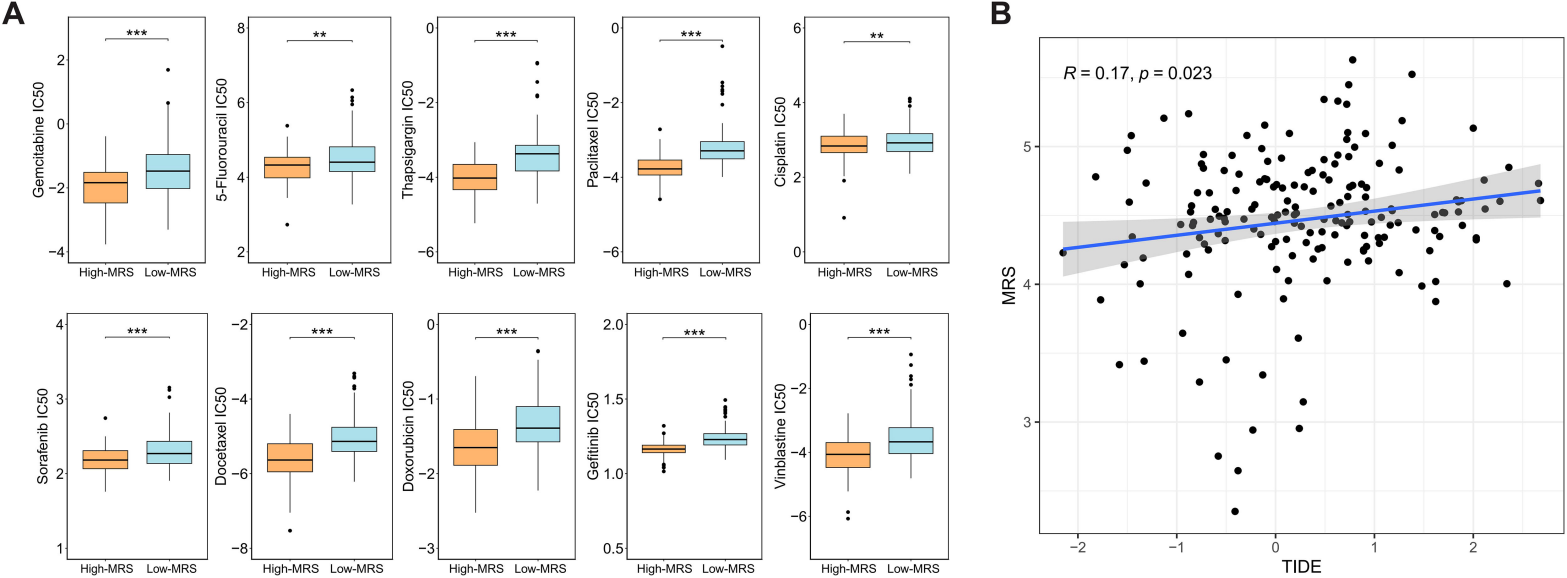
Therapeutic response based on MRS. **(A)** Comparison of drug sensitivity between high- and low-MRS groups across ten chemotherapy drugs. **(B)** Correlation between MRS and TIDE scores. **p < 0.01, ***p < 0.001.

In order to identify potential therapeutic agents, we compared chemotherapy drug sensitivity between high-MRS and low-MRS groups in TCGA-PAAD cohort. Analyses demonstrated that ten chemotherapy agents, including gemcitabine, 5-fluorouracil, thapsigargin, paclitaxel, cisplatin, sorafenib, docetaxel, doxorubicin, gefitinib, vinblastine, showed significantly lower IC50 values in high-MRS group, indicating their potential therapeutic efficacy for high-MRS patients (P < 0.05, **Figure 7A**). MRS showed a positive correlation with TIDE scores, suggesting that patients with lower MRS may have a higher likelihood of benefiting from immunotherapy (P = 0.023, **Figure 7B**). Furthermore, the DSigDB database was used to predict candidate drugs associated with the three core MRGs. The top 10 predicted drug compounds are presented in **Table 1**.

**Table 1:** Top 10 predicted drug compounds.

Term	P-value	Adjusted P-value	Combined Score	Genes
Linsidomine CTD 00000073	3.79E-06	0.001444848	23747.75571	CAT;PRKN
INDIRUBIN-3’-MONOXIME CTD 00004181	2.22E-05	0.003595141	8063.260471	CAT;PRKN
dopamine CTD 00005870	2.83E-05	0.003595141	6959.580823	CAT;PRKN
apigenin CTD 00007429	9.29E-05	0.00884862	3356.925155	CAT;PRKN
lindane CTD 00001115	1.29E-04	0.009839664	2736.968135	CEBPB;CAT
MG-132 CTD 00002789	2.22E-04	0.012812811	1950.89639	CEBPB;PRKN
1,9-Pyrazoloanthrone CTD 00003948	2.43E-04	0.012812811	1842.936118	CEBPB;CAT
Capsaicin CTD 00005570	3.15E-04	0.012812811	1564.86598	CEBPB;CAT
Cube root extract CTD 00006707	3.30E-04	0.012812811	1517.970575	CAT;PRKN
PARAQUAT CTD 00006471	3.36E-04	0.012812811	1499.895828	CEBPB;CAT

### Construction and validation of the prognostic nomogram

**Figure 8:**
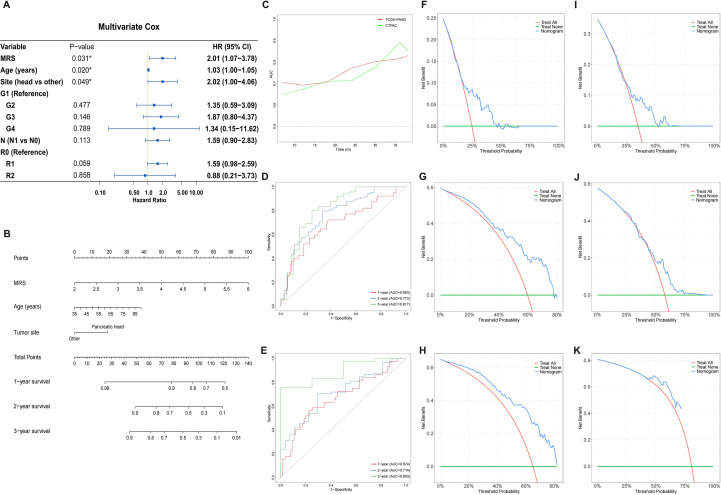
Development and validation of prognostic nomogram. **(A)** Forest plot of multivariable Cox regression analysis incorporating MRS and clinicopathological parameters. **(B)** A prognostic nomogram for predicting 1-, 2-, and 3-year OS in PC patients. **(C)** Time-dependent ROC curves in the training cohort (TCGA-PAAD cohort) and validation cohort (CPTAC-PDAC cohort). **(D, E)** ROC curves evaluating 1-, 2-, and 3- year OS prediction by the nomogram in the training cohort **(D)** and validation cohort**(E)**. **(F–H)** The DCA of nomogram for 1-year **(F)**, 2-year **(G)**, and 3-year **(H)** OS in the training cohort. **(I–K)** The DCA of nomogram for 1-year **(I)**, 2-year **(J)**, and 3-year **(K)** OS in the validation cohort.

To enhance the prognostic accuracy for PC patients, a nomogram was constructed by integrating MRS with clinicopathological features. Initial univariable Cox regression analysis of prognostic factors was conducted through the TCGA-PAAD cohort as the training set, assessing MRS and routine clinicopathological variables (age, sex, tumor site, T stage, N stage, R0 resection, tumor stage, and pathological grade) (**Supplementary Figure 5**, **Supplementary Table 4**). Variables with a P-value <0.1 were subsequently incorporated into the multivariate Cox regression analysis, which ultimately identified age, tumor site and MRS as independent prognostic factors for PC patients (P < 0.05, **Figure 8A**, **Supplementary Table 4**). A nomogram incorporating these variables was constructed to predict the 1-, 2-, and 3-year OS of PC patients (**Figure 8B**). In order to validate its effectiveness and reliability, the CPTAC-PDAC cohort was employed as an external validation set. In both training and validation cohorts, time-dependent ROC analyses demonstrated that AUC values predominantly fluctuated above 0.7 (**Figure 8C**). The AUC values for 1-, 2-, and 3-year OS were 0.693, 0.773, and 0.817 in the training cohort (**Figure 8D**), and 0.674, 0.714, and 0.893 in the validation cohort (**Figure 8E**), respectively. These results indicate that the nomogram provided strong predictive accuracy for the 1-, 2-, and 3-year OS of PC patients. Furthermore, DCA was performed to quantify the nomogram’s clinical benefit. In both training and validation cohorts, the DCA for 1-, 2-, and 3-year OS demonstrated that the nomogram provided superior net clinical benefits within a reasonable threshold probability range, supporting its robust clinical applicability (**Figures 8F–K**).

### Experimental validation for potential mechanisms of UPR^mt^

**Figure 9:**
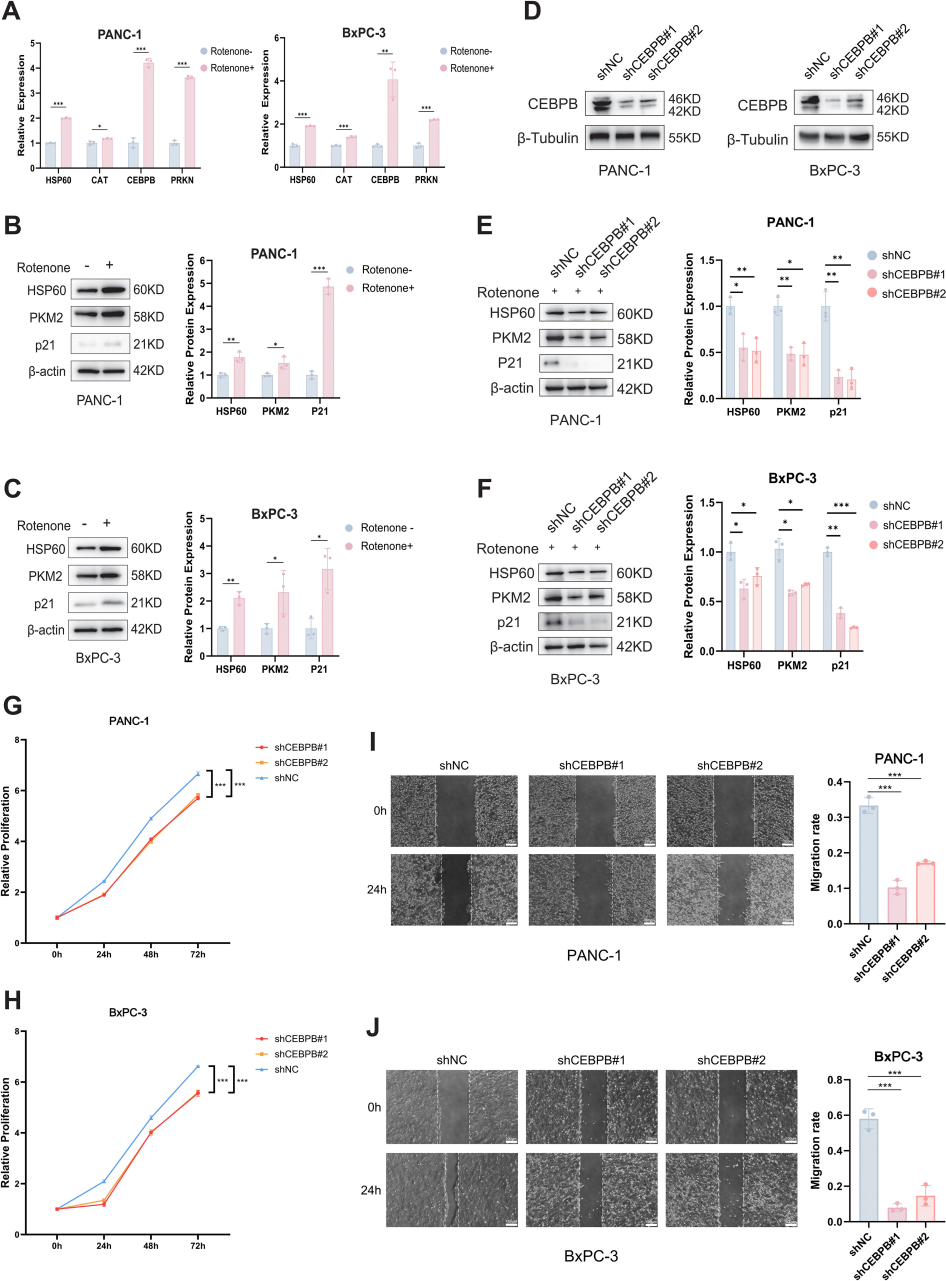
Experimental validation for potential mechanisms of UPR^mt^. **(A)** Quantitative RT-PCR analysis of core MRGs’ expression in PANC-1 and BxPC-3 cells following rotenone stimulation. **(B, C)** Western blot analysis showing the association between UPR^mt^ and both p53 and glycolysis pathways in PANC-1 **(B)** and BxPC-3 **(C)** cells. **(D)** Validation of CEBPB silencing efficiency in PANC-1 and BxPC-3 cells. **(E, F)** Changes of UPR^mt^, p53, and glycolysis pathway activities in PANC-1 **(E)** and BxPC-3 **(F)** cells after CEBPB knockdown under rotenone stimulation. **(G–H)** CCK-8 assay showing the proliferative capacity of PANC-1 **(G)** and BxPC-3 **(H)** cells after CEBPB knockdown. **(I, J)** Wound healing assay evaluating the migration of PANC-1 **(I)** and BxPC-3 **(J)** cells after CEBPB knockdown. Scale bar = 100 μm. *p < 0.05, **p < 0.01, ***p < 0.001.

The association between core MRGs and UPR^mt^ was further validated through *in vitro* experiments, and the oncogenic mechanisms of UPR^mt^ were investigated. Rotenone was employed to induce mitochondrial stress and trigger UPR^mt^. As a core component of UPR^mt^, HSP60 (HSPD1 encoding) was employed here as a biomarker for UPR^mt^ activation. The results of qRT-PCR demonstrated that CAT, CEBPB, and PRKN were significantly upregulated under acute mitochondrial stress conditions (**Figure 9A**), thereby validating their association with UPR^mt^. The GSVA analysis above revealed concurrent upregulation of both the “P53_PATHWAY” and “GLYCOLYSIS” pathways in both C2 and High-MRS group (**Figures 2G**, **6H**), suggesting potential crosstalk between UPR^mt^ and them. Western blot demonstrated synchronous upregulation of HSP60 (a marker of UPR^mt^), p21 (a marker of p53 pathway), and PKM2 (a marker of glycolysis) under mitochondrial stress condition(**Figures 9B, C**), supporting the above inference. To further validate the crosstalk among the UPR^mt^, glycolysis, and p53 pathways, the core MRG CEBPB was silenced (**Figure 9D**) and the mitochondrial stress responses were re-evaluated. Upon rotenone-induced mitochondrial stress, knockdown of CEBPB resulted in decreased expression of HSP60, PKM2, and p21, indicating reduced activity of the UPR^mt^, glycolysis, and p53 pathways (**Figures 9E, F**). CCK-8 assays demonstrated that silencing CEBPB significantly suppressed the proliferation of PANC-1 and BxPC-3 cells (**Figures 9G, H**), while wound healing assays revealed a significant reduction in their migratory capacity (**Figures 9I, J**). Collectively, these findings suggest that CEBPB contributes to the malignant progression of PC by promoting both proliferation and migration.

## Discussion

Mounting evidence in recent years has revealed a close association between the UPR^mt^ and cancer (20, 44). Mitochondrial stress is frequently elevated in cancer cells, and the UPR^mt^ acts as a protective mechanism to sustain mitochondrial homeostasis, thereby facilitating tumor progression (45, 46). Previous studies demonstrated that UPR^mt^ activation significantly enhances tumor initiation and invasiveness in mice models (47). As highlighted by Zhang et al. (19), various oncogenic factors and UPR^mt^-related pathways could reinforce each other. Consequently, targeting UPR^mt^ is increasingly considered a promising therapeutic approach in oncology. At present, comprehensive bioinformatic analyses of UPR^mt^ remain relatively scarce. To the best of our knowledge, our study is the first investigation that comprehensively analyzes the functional roles of MRGs in PC and establishes an UPR^mt^-related prognostic model.

This study systematically interrogated 43 MRGs through multidimensional analysis encompassing expression profiles, prognostic value, immune infiltration features, and drug sensitivity prediction, to delineate the role of UPR^mt^ in PC pathogenesis. First, we stratified PC patients into molecular subtypes through consensus clustering of MRG expression profiles, and subsequently assessed immune microenvironment heterogeneity across subtypes. Then, by leveraging three machine learning algorithms (LASSO, RSF, and XGBoost) along with univariate and multivariate Cox regression analyses, three core prognostic MRGs were identified. Next, we constructed an MRS using these three core MRGs, and further investigated its oncogenic mechanisms while predicting for potential therapeutic drugs. Finally, we developed an MRS-based nomogram and validated its predictive accuracy across multiple independent cohorts.

Through multi-step screening, we finally identified three core prognostic MRGs—CAT, CEBPB, and PRKN. CAT (Catalase), a critical antioxidant enzyme, sustains cellular redox equilibrium by catalytically degrading hydrogen peroxide (48, 49). In the hypoxic tumor microenvironment, CAT enables tumor cells to resist ROS-induced apoptosis and buffers mitochondrial oxidative stress, thereby facilitating tumor progression (50). CEBPB (CCAAT enhancer binding protein beta) is a key transcriptional regulator of immune and inflammatory responses, and plays a critical role in regulating the UPR^mt^ triggered by the accumulation of unfolded proteins within the mitochondrial matrix (15, 51). Previous studies have reported that CEBPB is highly expressed in PC tissues, and this upregulation is significantly associated with a poor prognosis (52, 53). PRKN (Parkin RBR E3 ubiquitin-protein ligase) is a core participant in mitophagy, identifying and eliminating damaged mitochondria when mitochondrial proteotoxic stress exceeds the repair capacity of the UPR^mt^ (54, 55). It has been reported that PRKN serves as a protective prognostic factor in PC, where its downregulation promotes tumor progression and correlates with a poor clinical prognosis (55, 56). Based on these three key MRGs, we constructed a novel MRS to predict the OS of PC patients. The MRS demonstrated robust prognostic accuracy in the training cohort and two independent validation cohorts. We employed rotenone to induce acute mitochondrial stress and trigger UPR^mt^, and observed UPR^mt^ activation accompanied by upregulated expression of three core MRGs. These findings corroborate the association between MRGs and UPR^mt^, thereby strengthening the validity of the MRS. To validate the functional role of the core MRGs in driving PC malignancy, we silenced CEBPB and observed a significant reduction in the proliferation and migration of PC cells. Furthermore, we enhanced predictive accuracy by integrating the MRS with clinicopathological features into a nomogram, which reliably estimated 1-, 2-, and 3-year OS in PC patients.

PC exhibits an exceptionally poor response to immunotherapy, and effective immunotherapeutic strategies remain lacking for this malignancy (57). To evaluate the influence of MRGs on the immune microenvironment, we classified PC patients into two distinct clusters using consensus clustering. The two clusters exhibited significantly different immune infiltration patterns. Patients with high UPR^mt^ activity (C2) exhibited lower infiltration of various immune cells, including B cells, CD8+ T cells, and macrophages, indicating a “cold” immune microenvironment that is consistent with their poorer prognosis. Moreover, a positive correlation was observed between the MRS and TIDE score, suggesting that patients with higher MRS may derive less benefit from immunotherapy. Similarly, Qian et al. (58) reported that elevated UPR^mt^ levels correlate with diminished immune infiltration in lung adenocarcinoma. These findings imply that UPR^mt^ may mediate immunosuppression through potential mechanisms such as modulating T cell-related cytokines, though this warrants further investigation. Previous studies have shown that UPR^mt^ can promote the formation of a locally immunosuppressive microenvironment via metabolic reprogramming (59), highlighting the therapeutic potential of combining immunotherapy with metabolic-targeting agents for PC. Notably, the two clusters exhibited distinct immune checkpoint expression profiles. For instance, TGFB1 exhibited significant upregulation in C2, which suggests that patients with enhanced UPR^mt^ activity may exhibit higher response rates to transforming growth factor-beta (TGF-beta) inhibition therapy. As a critical regulator of epithelial-mesenchymal transition and immunosuppressive microenvironment in PC, TGF-beta has emerged as a promising therapeutic target in recent years (60, 61). Beyond TGFB1, other immune checkpoints such as TIGIT, CD276, and PVR also showed significantly differential expression between clusters C1 and C2. This distinct immune checkpoint expression profile may provide individualized immunotherapies for PC patients.

PC often exhibits a pronounced propensity for chemotherapy resistance and lacks effective targeted therapies (62). Based on MRS, we conducted a drug sensitivity prediction to explore personalized therapeutic strategies for PC patients. Drug sensitivity profiling across 10 common chemotherapeutic drugs (gemcitabine, 5-fluorouracil, thapsigargin, paclitaxel, cisplatin, sorafenib, docetaxel, doxorubicin, gefitinib, vinblastine) demonstrated that PC patients with high MRS displayed significantly reduced IC50 values relative to those with low MRS. Among these agents, gemcitabine and 5-fluorouracil are first-line chemotherapeutic drugs for PC, while thapsigargin serves as an inducer for unfolded protein response. These results provide potential references for individualized treatment of PC patients. Consistent with our results, Zhang et al. (21) have revealed that hepatocellular carcinoma patients in high MRGs expression clusters exhibited significantly lower IC50 values for common chemotherapeutic agents relative to those in low MRGs expression clusters. Liu et al. (22) similarly observed that reduced ATF5 (an important UPR^mt^ gene) expression correlated with increased IC50 values to cisplatin, paclitaxel, and etoposide in cervical squamous cell carcinoma and endocervical adenocarcinoma cells. However, the observed higher drug sensitivity in high-risk patients appears counter-intuitive. This contradiction suggests that the relationship between UPR^mt^ and drug sensitivity is not simply linear, but likely involves more complex interactions. A recent study reported that sorafenib treatment induces significant downregulation of the majority of core prognostic MRGs in hepatocellular carcinoma cells (21). It is noteworthy that predictions from bioinformatics analyses reflect the intrinsic drug sensitivity of tumor cells under idealized and controlled *in vitro* conditions, which cannot fully account for the impacts of tumor heterogeneity and microenvironment. Therefore, further validation of this finding in *in vivo* models is required. Moreover, we predicted potential therapeutic drugs targeting the three core MRGs using the DSigDB database, a comprehensive resource linking drugs to gene expression signatures derived from multiple public databases. Based on the combined scores with the core MRGs, we listed the top 10 candidate compounds to facilitate future development of UPR^mt^-targeted therapies.

To further investigate the oncogenic mechanisms of UPR^mt^ in PC, we analyzed the activity of 50 Hallmark pathways across distinct clusters and MRS-based subgroups. Notably, GSVA analysis revealed concurrent upregulation of “P53 PATHWAY” and “GLYCOLYSIS” pathways in both C2 and High-MRS groups, suggesting a potential interplay among mitochondrial stress, oncogenic signaling, and metabolic reprogramming. Previous studies have reported that oncogenic signaling and metabolic abnormalities in tumors could disrupt the microenvironment and impair mitochondrial function, thereby activating the UPR^mt^ (21). Given the exceptionally high frequency of P53 mutations in PC, this enrichment likely does not reflect canonical tumor-suppressive functions of wild-type P53. Instead, it may indicate aberrant reprogramming of P53-associated networks driven by mutant alleles. Mutant P53 has been shown to enhance glycolytic flux by upregulating key glycolytic enzymes, thereby reinforcing the metabolic shift toward glucose dependence (63–65). Moreover, studies have reported that under stress conditions, the UPR suppresses oxidative phosphorylation while upregulating glycolysis (66). This pattern aligns with our observed glycolysis enrichment in both the C2 and high-MRS groups. Subsequent western blot analysis further validated concurrent upregulation of HSP60 (a UPR^mt^ marker), p21 (a p53 pathway marker), and PKM2 (a glycolytic marker) under UPR^mt^ activation. Silencing the core MRG CEBPB markedly reduced the protein levels of these molecules, indicating that CEBPB plays a crucial regulatory role in this process. These findings reveal a previously unrecognized functional crosstalk among the UPR^mt^, glycolysis, and p53 signaling pathways, suggesting that UPR^mt^ regulates both proliferation and metabolic reprogramming in PC cells, providing a valuable reference for further investigation into the oncogenic mechanisms of UPR^mt^.

Recent studies have reported several prognostic models associated with mitochondrial homeostasis (21, 56). Zhuo et al. (56) developed a 3-gene prognostic model for pancreatic cancer based on mitophagy, another critical aspect of mitochondrial homeostasis. Zhang et al. (21) established a UPR^mt^-related 7-gene prognostic model for hepatocellular carcinoma. These models demonstrated good performance in predicting patient survival outcomes. Compared with existing models, our model offers several advantages. First, it is the first UPR^mt^-related prognostic model for PC. Second, we integrated multiple machine learning algorithms for gene selection to enhance the robustness and predictive accuracy. Third, DCA was employed to assess the clinical benefits of our predictive model, demonstrating its clinical applicability.

This study has several limitations that should be acknowledged. First, both the training and validation cohorts were derived from public databases, and thus, further large-scale prospective multicenter studies are needed to validate our findings. Second, while we conducted preliminary experimental verification of the core MRGs, their regulatory mechanisms in PC progression remain to be fully elucidated. Additionally, the proposed therapeutic strategy requires rigorous clinical validation. Future research should focus on multicenter prospective studies to further evaluate the predictive performance of the model and facilitate its iterative refinement.

## Conclusion

In summary, this study offers a comprehensive evaluation of MRGs in PC, encompassing their expression profiles, prognostic significance, immunomodulatory effects, and drug sensitivity. By employing machine learning algorithms, we identified three core prognostic MRGs (CAT, CEBPB, and PRKN) and subsequently developed a novel MRS to predict patients’ survival and treatment responsiveness. The MRS-based prognostic nomogram demonstrated robust predictive accuracy for 1-, 2-, and 3-year OS of PC patients, with consistent validation across multiple independent cohorts. Furthermore, we identified the potential associations between UPR^mt^ and both the p53 pathway and glycolysis, thereby providing novel insights into the oncogenic mechanisms of UPR^mt^. Our findings elucidate the critical role of the UPR^mt^ in PC progression, providing valuable references for individualized therapeutic strategies and prognostic assessment in clinical management.

## Data Availability

The original contributions presented in the study are included in the article/**Supplementary Material**. Further inquiries can be directed to the corresponding authors.
