# Supplementary material for: Comprehensive analysis of mitochondrial unfolded protein response related genes for prognosis and therapeutic response in pancreatic cancer
Source: Front Immunol. 2026 Feb 5;17:1717925. doi: 10.3389/fimmu.2026.1717925 (PMC12916624; doi:10.3389/fimmu.2026.1717925)

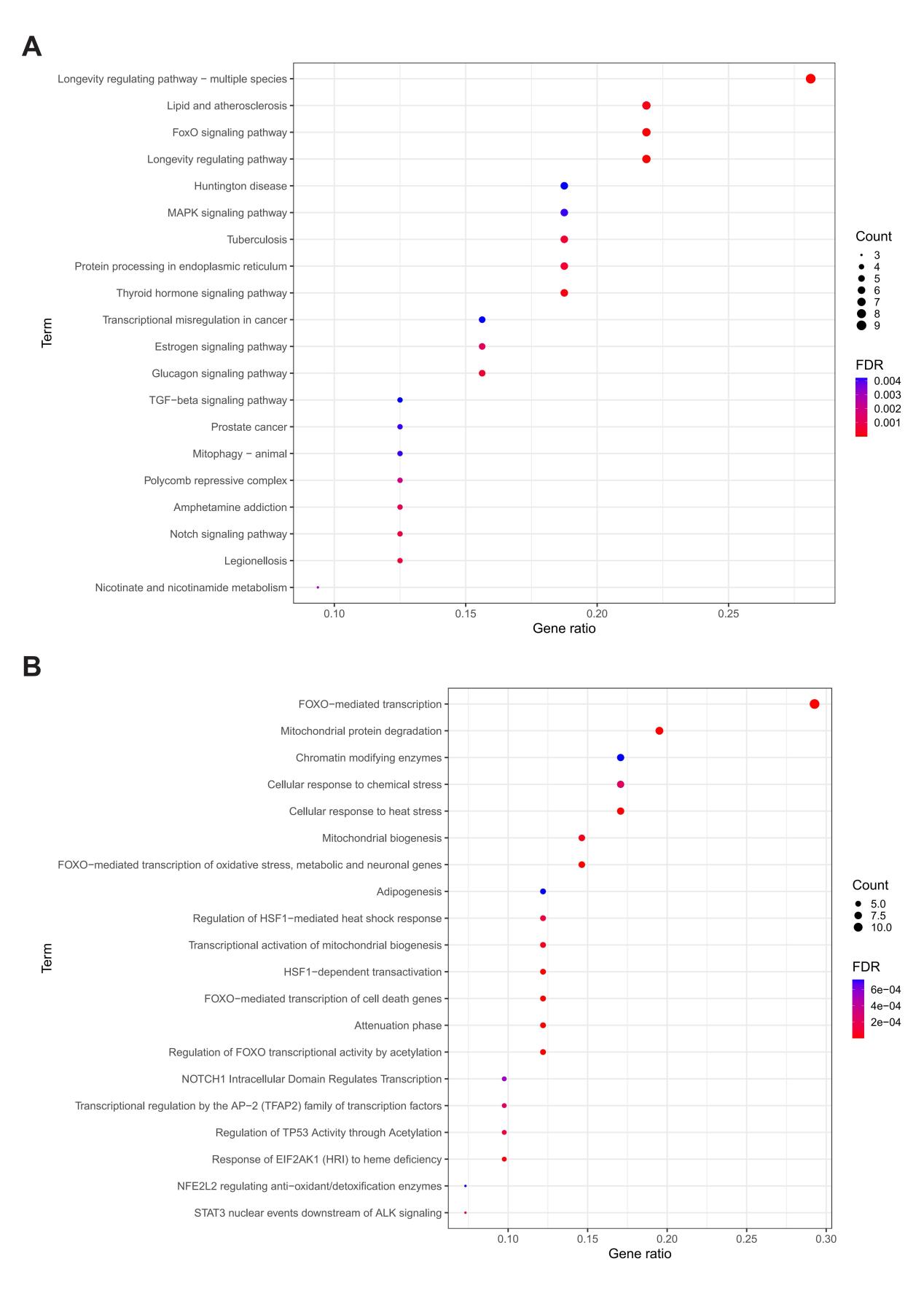
Supplementary Figure 1 KEGG (A) and Reactome (B) pathway enrichment analysis of MRGs.

Supplementary Figure
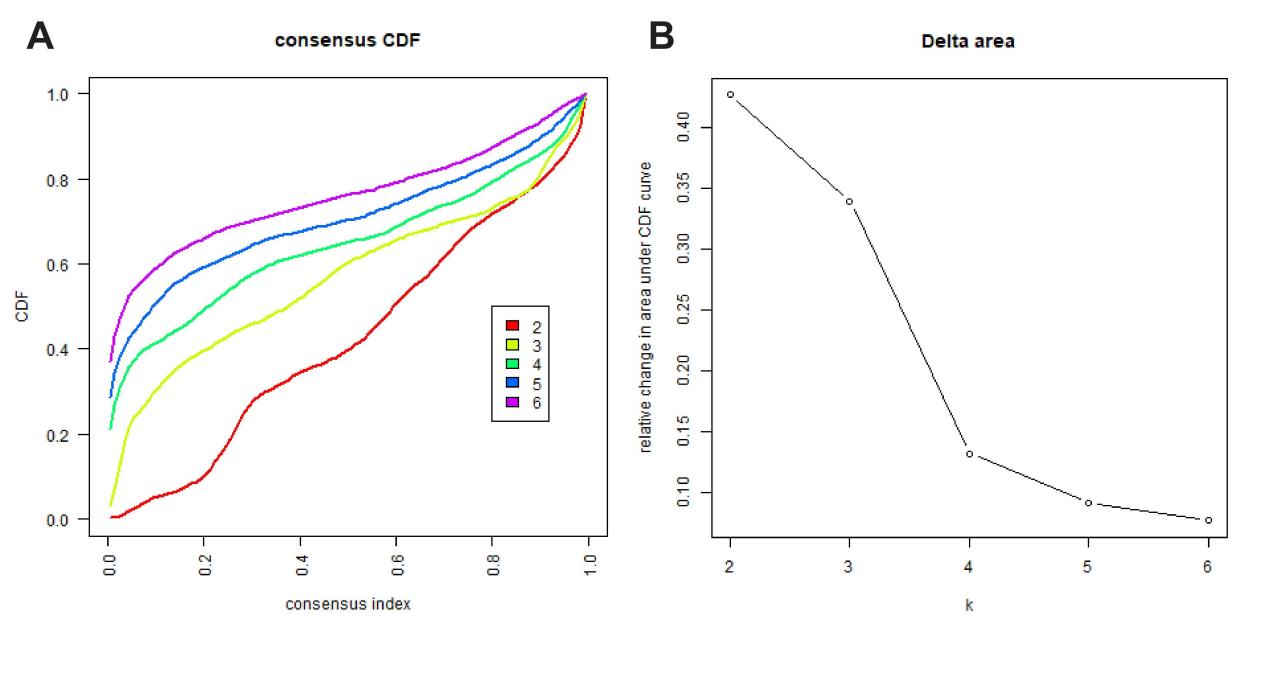
2 Consensus CDF plot (A) and delta area plot (B) of consensus clustering


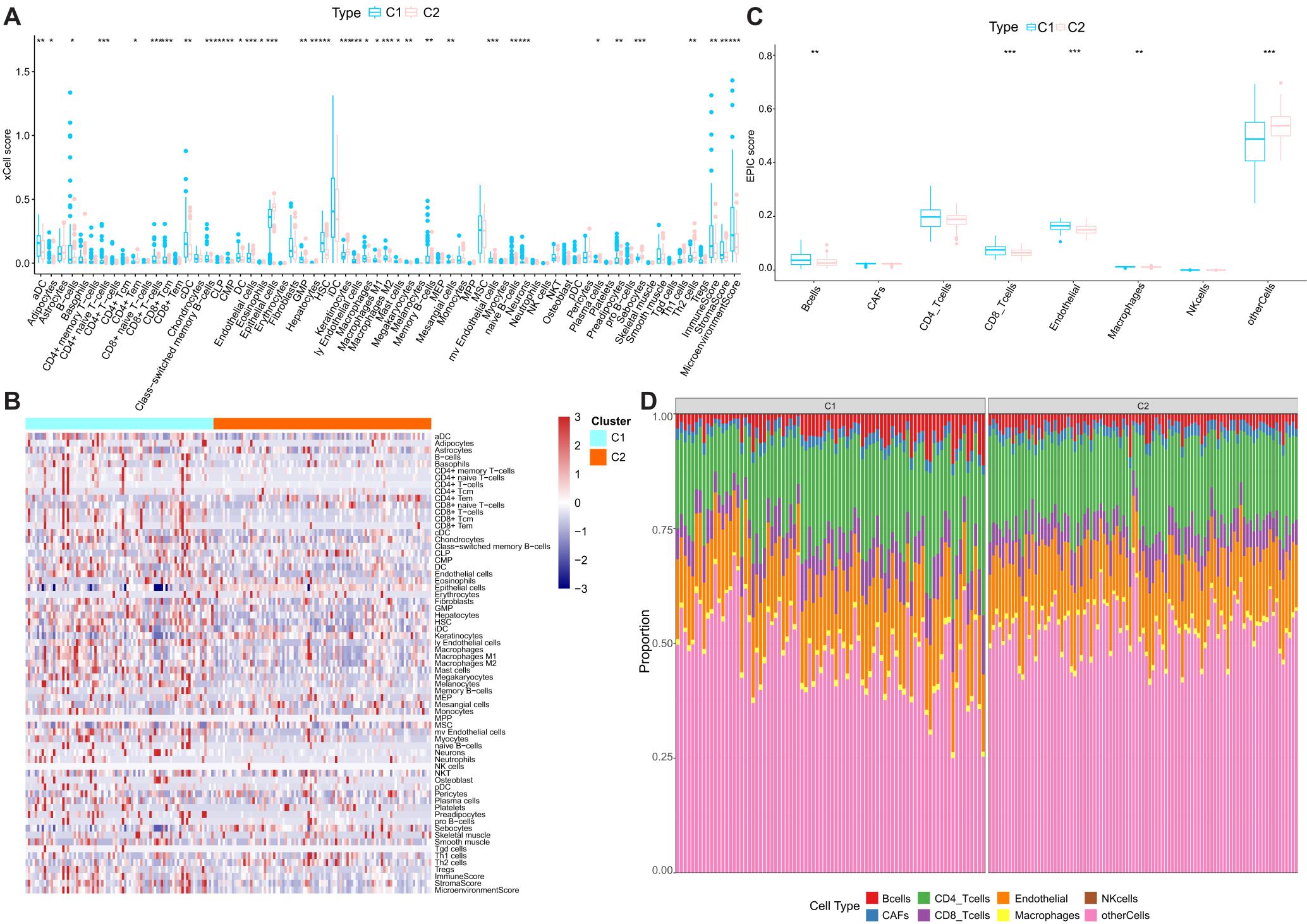


Supplementary Figure 3 Immunocyte infiltration analysis between two clusters

1. Box plot comparing immune infiltration levels between two clusters using xCell.
2. Heatmap of immune infiltration profiling based on xCell. (C) Box plot comparing immune infiltration levels between two clusters using EPIC. (D) Proportional infiltration of immune cells in each PC patient based on EPIC.



Supplementary Figure 4 Kaplan-Meier survival curves stratified by optimal cutoff value of MRS

(A) TCGA-PAAD cohort; (B) CPTAC-PDAC cohort; (C) GSE224564 cohort

Supplementary Figure 5 Forest plot of univariate Cox regression analysis incorporating MRS and clinicopathological features.
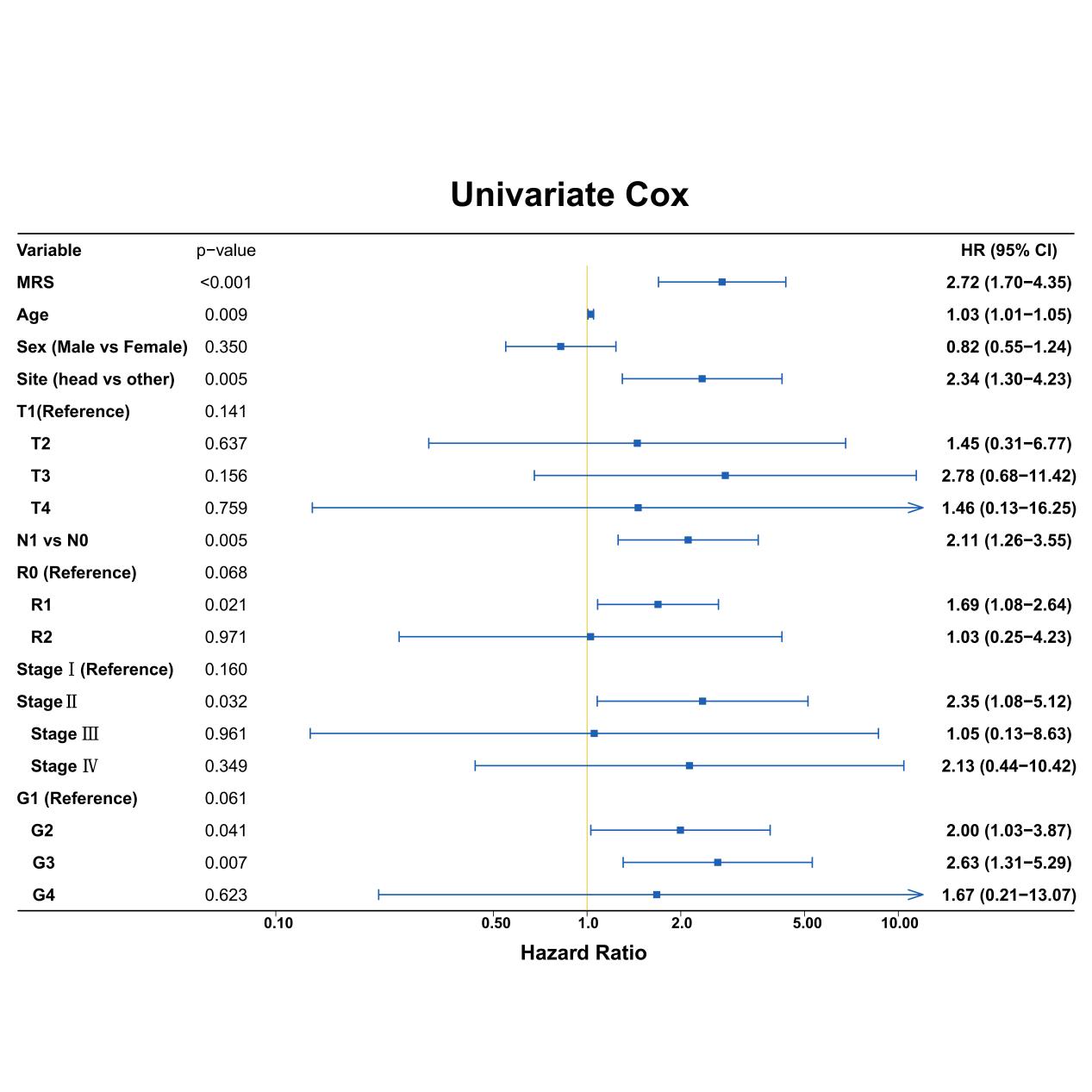

Supplement: Supplementary file 1 [file DataSheet1.docx]
