# Supplementary material for: Comprehensive analysis of mitochondrial unfolded protein response related genes for prognosis and therapeutic response in pancreatic cancer
Source: Front Immunol. 2026 Feb 5;17:1717925. doi: 10.3389/fimmu.2026.1717925 (PMC12916624; doi:10.3389/fimmu.2026.1717925)
Supplement: Supplementary file 2 [file Table1.docx]

| **Supplementary Table 1 Source of the datasets** | | | |
| --- | --- | --- | --- |
| Cohort | Source | Sample Size | Link |
| TCGA-PAAD | UCSC Xena | 177 tumor and 4 normal samples | http://xena.ucsc.edu/ |
| CPTAC-PDAC | LinkedOmics | 135 tumor and 21 normal samples | http://www.linkedomics.org/ |
| GSE224564 | GEO | 175 tumor samples | http://www.ncbi.nlm.nih.gov/geo |
| TCGA-TARGET-GTEx | UCSC Xena | 177 tumor and 171 normal samples | http://xena.ucsc.edu/ |
