# Supplementary material for: Comprehensive analysis of mitochondrial unfolded protein response related genes for prognosis and therapeutic response in pancreatic cancer
Source: Front Immunol. 2026 Feb 5;17:1717925. doi: 10.3389/fimmu.2026.1717925 (PMC12916624; doi:10.3389/fimmu.2026.1717925)
Supplement: Supplementary file 3 [file Table2.docx]

| **Supplementary Table 2** 43 mitochondrial unfolded protein response-related genes | |
| --- | --- |
| Gene symbol | Ensembl ID |
| ABCB10 | ENSG00000135776 |
| AKT1 | ENSG00000142208 |
| ATF4 | ENSG00000128272 |
| ATF5 | ENSG00000169136 |
| CAT | ENSG00000121691 |
| CEBPB | ENSG00000172216 |
| CLPP | ENSG00000125656 |
| CLPX | ENSG00000166855 |
| CREBBP | ENSG00000005339 |
| DDIT3 | ENSG00000175197 |
| DEFA5 | ENSG00000164816 |
| DNAJA1 | ENSG00000086061 |
| EHMT1 | ENSG00000181090 |
| EP300 | ENSG00000100393 |
| ESR1 | ENSG00000091831 |
| FGF21 | ENSG00000105550 |
| FOXO3 | ENSG00000118689 |
| GDF15 | ENSG00000130513 |
| HDAC1 | ENSG00000116478 |
| HDAC2 | ENSG00000196591 |
| HSF1 | ENSG00000185122 |
| HSPA1A | ENSG00000204389 |
| HSPA1B | ENSG00000204388 |
| HSPA9 | ENSG00000113013 |
| HSPD1 | ENSG00000144381 |
| HSPE1 | ENSG00000115541 |
| HTRA2 | ENSG00000115317 |
| KDM6B | ENSG00000132510 |
| LONP1 | ENSG00000196365 |
| LRPPRC | ENSG00000138095 |
| MRPS5 | ENSG00000144029 |
| NRF1 | ENSG00000106459 |
| PHF8 | ENSG00000172943 |
| PINK1 | ENSG00000158828 |
| PITRM1 | ENSG00000107959 |
| PRKN | ENSG00000185345 |
| PRORP | ENSG00000100890 |
| SIRT1 | ENSG00000096717 |
| SIRT3 | ENSG00000142082 |
| SIRT7 | ENSG00000187531 |
| SOD2 | ENSG00000112096 |
| SSBP1 | ENSG00000106028 |
| YME1L1 | ENSG00000136758 |
