# Supplementary material for: Comprehensive analysis of mitochondrial unfolded protein response related genes for prognosis and therapeutic response in pancreatic cancer
Source: Front Immunol. 2026 Feb 5;17:1717925. doi: 10.3389/fimmu.2026.1717925 (PMC12916624; doi:10.3389/fimmu.2026.1717925)
Supplement: Supplementary file 4 [file Table3.docx]

| **Supplementary Table 3** Sequence of primers | | |
| --- | --- | --- |
| Gene | Primers | Sequence |
| Hsp60(HSPD1)(human) | Forward | AGTGTTCAGTCCATTGTCCC |
|  | Reverse | TGACTGCCACAACCTGAAG |
| β-actin(human) | Forward | CCTGGACTTCGAGCAAGAGATGG |
|  | Reverse | CAGGAAGGAAGGCTGGAAGAGTG |
| PRKN(human) | Forward | GACAGCAGGAAGGACTCACC |
|  | Reverse | GCTGCACTGTACCCTGAGTT |
| CEBPB(human) | Forward | GACAAGCACAGCGACGAGTA |
|  | Reverse | CTTGAACAAGTTCCGCAGGG |
| CAT(human) | Forward | CTATCCTGACACTCACCGCC |
|  | Reverse | CCACCCTGATTGTCCTGCAT |
