# Supplementary material for: Comprehensive analysis of mitochondrial unfolded protein response related genes for prognosis and therapeutic response in pancreatic cancer
Source: Front Immunol. 2026 Feb 5;17:1717925. doi: 10.3389/fimmu.2026.1717925 (PMC12916624; doi:10.3389/fimmu.2026.1717925)
Supplement: Supplementary file 5 [file Table4.docx]

| **Supplementary Table 4** Univariate and multivariate Cox regression analyses of MRS and clinicopathological features | | | | | | | |
| --- | --- | --- | --- | --- | --- | --- | --- |
| variable | Univariate cox | | |  | Multivariate cox | | |
|  | HR | 95%CI | P |  | HR | 95%CI | P |
| MRS | 2.716 | 1.696-4.350 | <0.001 |  | 2.009 | 1.068-3.781 | 0.031 |
| Age | 1.028 | 1.007-1.049 | 0.009 |  | 1.027 | 1.004-1.050 | 0.020 |
| Sex (male vs female) | 0.823 | 0.548-1.238 | 0.350 |  |  |  |  |
| Site (head vs other) | 2.343 | 1.298-4.230 | 0.005 |  | 2.019 | 1.003-4.064 | 0.049 |
| T1(Reference) |  |  | 0.141 |  |  |  |  |
| T2 | 1.450 | 0.31-6.773 | 0.637 |  |  |  |  |
| T3 | 2.780 | 0.677-11.421 | 0.156 |  |  |  |  |
| T4 | 1.459 | 0.131-16.255 | 0.759 |  |  |  |  |
| N（N1vs N0） | 2.112 | 1.258-3.547 | 0.005 |  | 1.594 | 0.896-2.833 | 0.113 |
| R0 (Reference) |  |  | 0.068 |  |  |  | 0.151 |
| R1 | 1.690 | 1.081-2.643 | 0.021 |  | 1.595 | 0.983-2.588 | 0.059 |
| R2 | 1.026 | 0.249-4.227 | 0.971 |  | 0.876 | 0.206-3.730 | 0.858 |
| Stage Ⅰ (Reference) |  |  | 0.160 |  |  |  |  |
| Stage Ⅱ | 2.350 | 1.078-5.125 | 0.032 |  |  |  |  |
| Stage Ⅲ | 1.054 | 0.129-8.633 | 0.961 |  |  |  |  |
| Stage Ⅳ | 2.133 | 0.437-10.416 | 0.349 |  |  |  |  |
| G1 (Reference) |  |  | 0.061 |  |  |  | 0.418 |
| G2 | 1.995 | 1.028-3.874 | 0.041 |  | 1.350 | 0.590-3.091 | 0.477 |
| G3 | 2.629 | 1.306-5.291 | 0.007 |  | 1.874 | 0.804-4.369 | 0.146 |
| G4 | 1.674 | 0.214-13.072 | 0.623 |  | 1.343 | 0.155-11.624 | 0.789 |

MRS, mitochondrial unfolded protein response related gene risk scores; HR, hazard ratio; CI, confidence interval.
